# Carboplatin-Induced Thrombocytopenia through JAK2 Downregulation, S-Phase Cell Cycle Arrest and Apoptosis in Megakaryocytes

**DOI:** 10.3390/ijms23116290

**Published:** 2022-06-03

**Authors:** Yi-Hong Wu, Hsing-Yu Chen, Wei-Chin Hong, Chen-Ying Wei, Jong-Hwei Su Pang

**Affiliations:** 1 Department of Chinese Medicine, Chang Gung Memorial Hospital, Guishan, Taoyuan 333, Taiwan; mzpjih@adm.cgmh.org.tw (Y.-H.W.); b8705016@gmail.com (H.-Y.C.); cute09272006@gmail.com (W.-C.H.); hedywei@cgmh.org.tw (C.-Y.W.); 2 School of Traditional Chinese Medicine, Chang Gung University, Guishan, Taoyuan 333, Taiwan; 3 Graduate Institute of Clinical Medical Sciences, Chang Gung University, Guishan, Taoyuan 333, Taiwan; 4 Department of Physical Medicine and Rehabilitation, Chang Gung Memorial Hospital, Guishan, Taoyuan 333, Taiwan

**Keywords:** carboplatin, thrombocytopenia, megakaryocyte, cell cycle, apoptosis, JAK2

## Abstract

Chemotherapy-induced thrombocytopenia (CIT) is a common complication when treating malignancies with cytotoxic agents wherein carboplatin is one of the most typical agents causing CIT. Janus kinase 2 (JAK2) is one of the critical enzymes to megakaryocyte proliferation and differentiation. However, the role of the JAK2 in CIT remains unclear. In this study, we used both carboplatin-induced CIT mice and MEG-01 cell line to examine the expression of JAK2 and signal transducer and activator of transcription 3 (STAT3) pathway. Under CIT, the expression of JAK2 was significantly reduced in vivo and in vitro. More surprisingly, the JAK2/STAT3 pathway remained inactivated even when thrombopoietin (TPO) was administered. On the other hand, carboplatin could cause prominent S phase cell cycle arrest and markedly increased apoptosis in MEG-01 cells. These results showed that the thrombopoiesis might be interfered through the downregulation of JAK2/STAT3 pathway by carboplatin in CIT, and the fact that exogenous TPO supplement cannot reactivate this pathway.

## 1. Introduction

Chemotherapy-induced thrombocytopenia (CIT) is one of the most common complications when treating cancers using cytotoxic agents (especially carboplatin-based regimens). About one-fifth cancer patients may suffer from treatment-related thrombocytopenia, and carboplatin is one of the most relevant pharmacologic factors [1]. Thrombocytopenia may cause higher risks of bleeding events, including gastrointestinal and hemorrhagic stroke, that may increase the cost, prolong hospital stay, and may even threaten chances of survival [2]. In most circumstances, a reduction in carboplatin dose, replacing carboplatin with other agents, and extending the interval between chemotherapies would be necessary if thrombocytopenia persists [1]. In addition to the direct cause of death from complications of thrombocytopenia, the lower dosage and delayed schedule of chemotherapy may compromise the survival benefit [3,4]. On the other hand, such dose reductions support the clinical need for effective thrombopoietic agents to treat or prevent CIT and allow maintenance of chemotherapy dose density and intensity. Nevertheless, despite the high prevalence of CIT, the understanding of management for CIT is still limited.

Many cytotoxic agents are known to cause thrombocytopenia by inducing hypoplasia of the bone marrow megakaryocytic cells [5,6,7]. For example, alkylating agents affect hematopoietic cells in the bone marrow [8], cyclophosphamide affects megakaryocyte progenitors instead of pluripotent stem cells [9], bortezomib prevents platelet release from megakaryocytes by inhibiting nuclear factor kappa B (NF-κB) [10], and some treatments promote platelet apoptosis. Besides, platelet homeostasis depends on the balance between B-cell lymphoma-extra large (Bcl-x(L)), the anti-apoptotic factor, and pro-apoptotic factors, Bcl-2 associated X protein (Bax) and Bcl-2 homologous antagonist/killer (Bak) [11,12]. Some chemotherapy agents may increase platelet destruction instead of production by decreasing the activity of Bcl-x(L) [13]. Cisplatin, which shares a similar structure with carboplatin, may also cause myelosuppression although in less extent than carboplatin [14]. Several possible pathogeneses have been proposed, such as inducing DNA damage in hematopoietic cells, changes in cytokines needed to cell growth, cell cycle arrest, or imbalance between Bcl-X(L), Bax, and BAK [14]. Since not all chemotherapy agents cause thrombocytopenia via the same pathway, and carboplatin induces much more severe myelosuppression effect than cisplatin [7], the study about the influence of carboplatin on megakaryocytes are still needed.

Janus kinase 2 (JAK2) is proposed as one of the crucial factors to the survival of megakaryocytes in addition to Protein kinase B (AKT) and phosphoinositide-3-kinase (PI3K) pathway [15]. Many cytokines require JAK2 for subsequent mitogenic signaling in hematopoietic cells, including erythropoietin and thrombopoietin (TPO). TPO is the most important endogenous cytokine to regulate megakaryopoiesis and thrombopoiesis that contributes to platelet production [16]. To maintain megakaryocytes survival, TPO binds to its receptor c-Mpl on megakaryocytes, which activate JAK2, and then activate the signal transducer and activator of transcription (STAT) family proteins. The STAT-related transcriptional factors in the nucleus are then activated by intracellular localization of STAT-family proteins, which may prevent megakaryocytes apoptosis and regulate megakaryocyte production [16,17]. Besides, the increased expression of Bcl-X(L) is also dependent on TPO stimulation to achieve the anti-apoptosis effect on megakaryocytes via JAK/STAT and phosphoinositide 3-kinase (PI3K) pathways [18]. For this reason, we proposed that the expression of JAK2 may determine the survival of megakaryocytes under the influence of cytotoxic agents, such as carboplatin. 

This study aimed to examine the influences of carboplatin on megakaryocytes. For this purpose, the relations between JAK2/STAT3 expression, cell cycle arrest and apoptosis of megakaryocyte and the CIT caused by carboplatin were explored both in vivo and in vitro. Additionally, to examine the effect of TPO under CIT, the expression of JAK2/STAT3 of megakaryocyte was examined subjected to carboplatin and subsequent TPO. The result of this study would broaden our understanding of the importance of JAK2/STAT3 pathway in CIT. 

## 2. Results

### 2.1. Carboplatin Induced Thrombocytopenia in SD Rats

**Figure 1:**
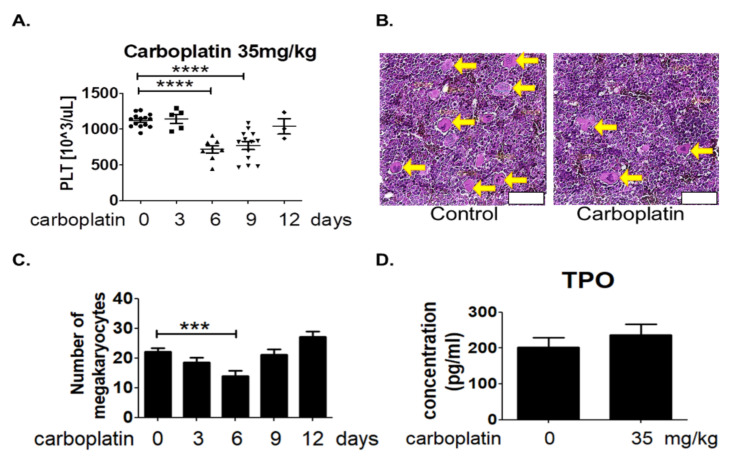
The time-dependent change of platelet counts in blood and megakaryocyte counts in bone marrow after carboplatin treatment. Rats were randomly divided into five groups (at least five rats in each group) and sacrificed at 0, 3, 6, 9, and 12 days after intraperitoneal injection of 35 mg/kg carboplatin. (**A**) The numbers of platelets in the blood were determined by blood analyzer at 0, 3, 6, 9 and 12 days after carboplatin treatment. (**B**) Photos shown were bone marrow sections prepared from control and carboplatin-treated rat at the sixth day. Megakaryocytes were identified based on their characteristic morphology such as large size, pink cytoplasm and lobated nucleus and indicated by the yellow arrows. Scale bar = 60 μm. (**C**) Megakaryocyte were counted in the HE-stained sections of bone marrow at 0, 3, 6, 9, and 12 days after carboplatin treatment under microscopic examination by Nikon ECLIPSE Ni-U. (**D**) TPO concentrations in the blood prepared from control and carboplatin-treated rat at the sixth day were measured by ELISA. (*** *p*-value < 0.001, **** *p*-value < 0.0001, three independent experiments were performed).

Rats with intraperitoneal injection of 35 mg/kg carboplatin were sacrificed at 0, 3, 6, 9, and 12 days after treatment. Results from the analysis of blood cells by automated hematology analyzer revealed the significantly decreased number of platelets in rat blood at the sixth day and slowly recovered from the 9th to 12th day after carboplatin treatment as shown in Figure 1A. About 34.6% decrease of the platelet number was found in rat received carboplatin treatment for six days as compared to control rat. However, there was no significant change in the number of other cell types including red blood cells and white blood cells (Table 1).

### 2.2. Carboplatin Significantly Reduced the Number of Megakaryocytes in Rat Bone Marrow

Since platelets are produced from megakaryocytes, it is possible that carboplatin might affect the number of megakaryocytes in bone marrow. Thigh bones were prepared from rats at 0, 3, 6, 9, and 12 days after intraperitoneal injection of 35 mg/kg carboplatin for histological examination by HE stains. Megakaryocyte was revealed by its uniquely large pinkish cytosol and lobated nucleus as indicated by yellow arrow in Figure 1B. The results in Figure 1C showed that the number of megakaryocytes in bone marrow after carboplatin treatment appeared to change in a U-shaped manner. A significant decrease was also found at the sixth day after carboplatin treatment which reasonably resulted in the reduction of platelets number in rat blood. Since the platelet formation is dependent on the TPO simulation, the concentration of TPO in blood was measured in rats with or without carboplatin treatment at the sixth day. However, result in Figure 1D showed no significant difference of TPO concentration in the blood between control and carboplatin group.

### 2.3. Carboplatin Induced Apoptosis of Megakaryocytes in Rat Bone Marrow

**Figure 2:**
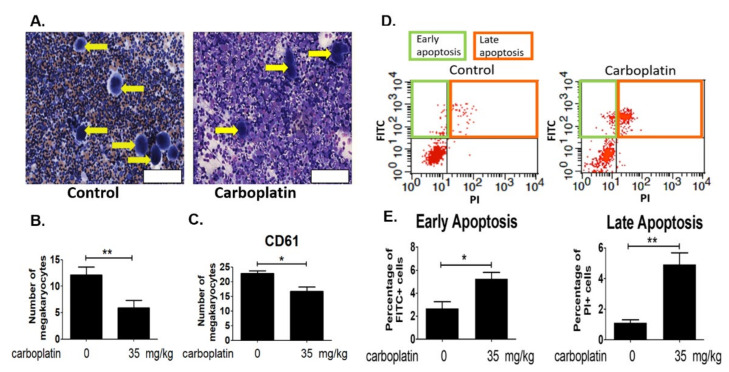
Number of megakaryocytes decreased after carboplatin treatment in vivo. Bone marrow washout from control and 35 mg/kg carboplatin-treated rat at the sixth day were used for the analysis. (**A**) After fixation and Liu’s staining, the megakaryocyte number was determined by counting. Scale bar = 120 μm. (**B**) Experiment was carried out in three different rats and result was statistically significant. (**C**) The CD61+ cells in the bone marrow washout were analyzed by flow cytometry. (**D**) A representative image from the flow cytometry was shown. Cells in the green square with the increased green fluorescence by the binding with FITC-conjugated annexin V were characterized as early apoptotic cells. On the other hand, cells in the red square with the increase of green fluorescence by the binding with FITC-conjugated annexin V and also the increase of red fluorescence from the PI inside the cytosol were characterized as late apoptotic cells. (**E**) Percentage of the early and late apoptotic cells in bone marrow washout was analyzed based on the results of flow cytometry. (* *p*-value < 0.05, ** *p*-value < 0.01, three independent experiments were performed).

Bone marrow cells were flushed out, fixed and revealed by Liu’s stain for the counting of megakaryocytes. Results in Figure 2A,B proved that carboplatin significantly decreased the number of megakaryocytes in bone marrow. The flushed out was also stained with megakaryocyte marker CD61 for the analysis by flow cytometry. Results in Figure 2C further confirmed the decrease of megakaryocytes in bone marrow after carboplatin treatment. Detection of apoptosis was carried out by staining the bone marrow cells with FITC-annexin V and propidium iodide. Results in Figure 2D showed that both the early (positive FITC-annexin-stained cells) and late apoptosis (positive propidium iodide-stained cells) were found to be increased in rat bone marrow after the carboplatin treatment.

### 2.4. Carboplatin Caused S-Phase Arrest and Apoptosis of Human Megakaryocytic Cells In Vitro

**Figure 3:**
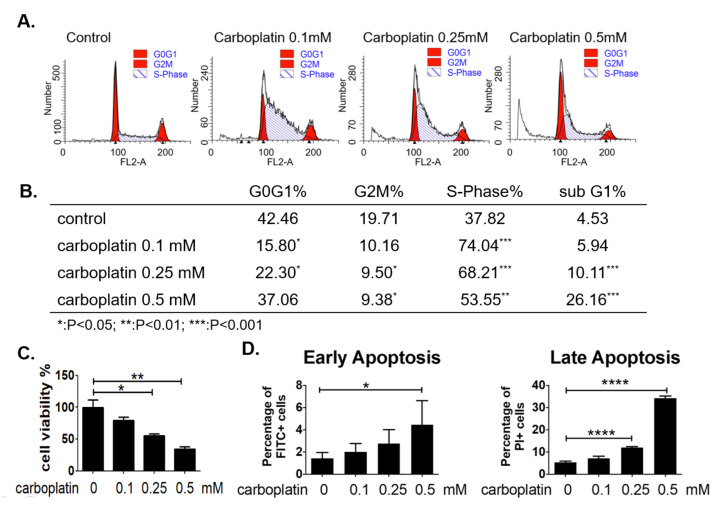
Carboplatin induced S-phase arrest and apoptosis of megakaryocytic cells dose-dependently in vitro. (**A**,**B**) MEG-01 cells were treated with different dose of carboplatin for 24 h and cell cycle distribution was analyzed by flow cytometry. (**C**) Cell viability of MEG-01 cells was determined at 24 h after carboplatin treatment by trypan blue exclusion assay. (**D**) Percentage of the early and late apoptotic cells was analyzed based on the results of flow cytometry. (* *p*-value < 0.05, ** *p*-value < 0.01, **** *p*-value < 0.0001, three independent experiments were performed).

MEG-01 cells were treated with carboplatin at concentration ranged from 0–0.5 mM for 24 h, and cell viability was determined by trypan blue exclusion assay. Result in Figure 3C showed that a dose-dependent decrease of cell viability was induced by carboplatin. The cell cycle distribution analyzed by flow cytometric method revealed a clear S-phase arrest of megakaryocytic cells by carboplatin as shown in Figure 3A,B. Cell apoptosis was also induced in megakaryocytic cells as revealed by the increase of subG1 cells after carboplatin treatment. The dose-dependent increase of apoptosis was correlated well with the decrease of S-phase arrested cells, suggesting higher concentration of carboplatin at 0.5 mM induced a significant amount of apoptosis mainly from the S-phase arrested cells (Figure 3B). In order to evaluate apoptosis using more appropriate marker, we have also stained cells using apoptosis detection kit containing FITC-annexin V and propidium iodide and analyzed by flow cytometry. Results in Figure 3D demonstrated the increase of both the early and late apoptosis after carboplatin treatment.

### 2.5. Carboplatin Modulated the Protein Expression of Cell Cycle-Dependent Genes and Cell Cycle Inhibitors

**Figure 4:**
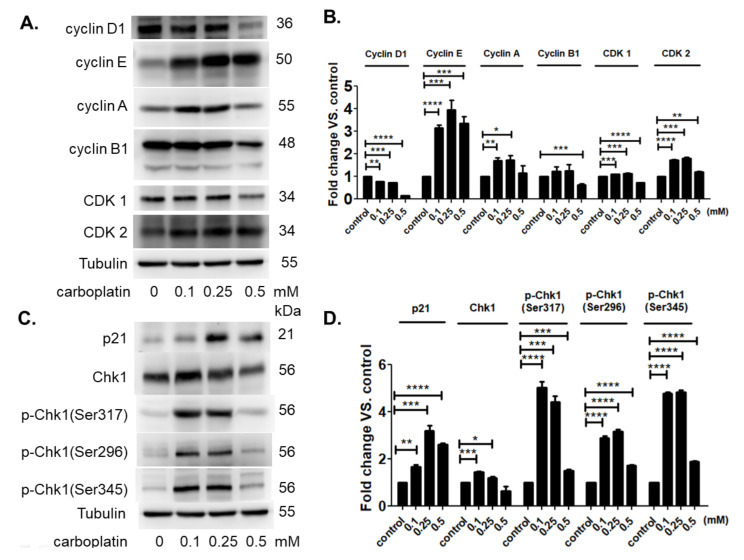
Carboplatin modulated the expressions of (**A**,**B**) cell cycle-dependent proteins and (**C**,**D**) cell cycle inhibitor proteins in megakaryocytic cells. MEG-01 cells were treated by carboplatin at different concentrations ranged from 0–0.5 mM for 24 h and protein expressions were then analyzed by western blot method. (* *p*-value < 0.05, ** *p*-value < 0.01, *** *p*-value < 0.001, **** *p*-value < 0.0001, three independent experiments were performed).

Carboplatin resulted in the S-phase arrest of human megakaryocytic cells at low concentration 0.1–0.25 mM. Decreased cyclin D expression correlated well with the decreased G1-phase. Increased cyclin E, A, and cyclin-dependent kinase 2 (CDK2) correlated well with increased S-phase. No significant change was detected in the expression of cyclin B and cyclin-dependent kinase 1 (CDK1). The expression of p21 was significantly induced in by carboplatin at low concentration 0.1–0.25 mM (Figure 4A,B). There was a slight increase in total amount of checkpoint kinase 1 (Chk1) at low concentration 0.1–0.25 mM. However, the activation of Chk1 was significantly found by the phosphorylation at ser296, ser317, and ser345, indicating a highly promoted activity of Chk1 that was correlated well with the S-phase arrest of megakaryocytic cells (Figure 4C,D). Carboplatin at high concentration 0.5 mM resulted in the marked increase of apoptosis in human megakaryocytic cells. The expression of all of these genes was apparently decreased or returned to control level except the expression of cyclin E which remained at high level (Figure 4A,B), suggesting a stable form of protein without appropriate degradation and the subsequent inhibition of cell cycle progression.

### 2.6. Carboplatin Suppressed the JAK2 Protein Expression Both In Vitro and In Vivo

**Figure 5:**
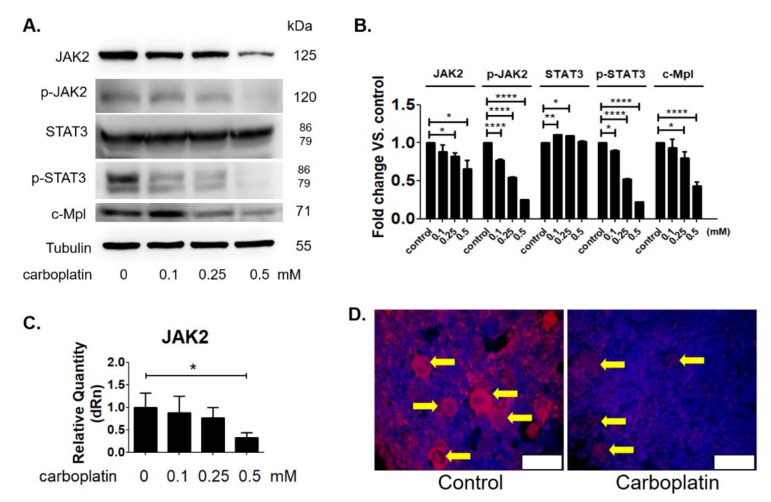
Carboplatin suppressed JAK2 and STAT3 activation in megakaryocytes. (**A**,**B**) MEG-01 cells were treated by carboplatin dose-dependently and the expressions of JAK2, p-JAK2, STAT3, p-STAT3 and c-Mpl were analyzed by western blot method and (**C**) the JAK2 mRNA expression was analyzed by RT/real-time PCR. (**D**) The JAK2 expression was analyzed in bone marrow sections prepared from control and carboplatin-treated rats at the sixth day by immunohistochemistry. Megakaryocytes were indicated by yellow arrow and the JAK2 expression was revealed by red fluorescent signal. Scale bar = 120 μm. (* *p*-value < 0.05, ** *p*-value < 0.01, **** *p*-value < 0.0001, three independent experiments were performed).

The JAK2 expression has been demonstrated to be tightly involved in the process of platelet production by megakaryocytes. Therefore, we next investigated the effect of carboplatin on the expression of JAK2 and the downstream signal pathway STAT3 activation. Figure 5A,B showed that the expression of JAK2, pJAK2 and STAT3 activation in human megakaryocytic cells was dose-dependently decreased by carboplatin treatment. The JAK2 mRNA expression was also inhibited by carboplatin in a dose-dependent manner (Figure 5C). As shown in Figure 5D, in vivo experiment also further confirmed the decrease of JAK2 expression of megakaryocytes in the bone marrow of rat treated by carboplatin for 6 days. The expression of c-Mpl, the receptor of TPO in megakaryocytic cells were also reduced by carboplatin as shown in Figure 5A,B.

### 2.7. TPO Could Not Activate the JAK2 Signal Pathway in the Presence of Carboplatin

**Figure 6:**
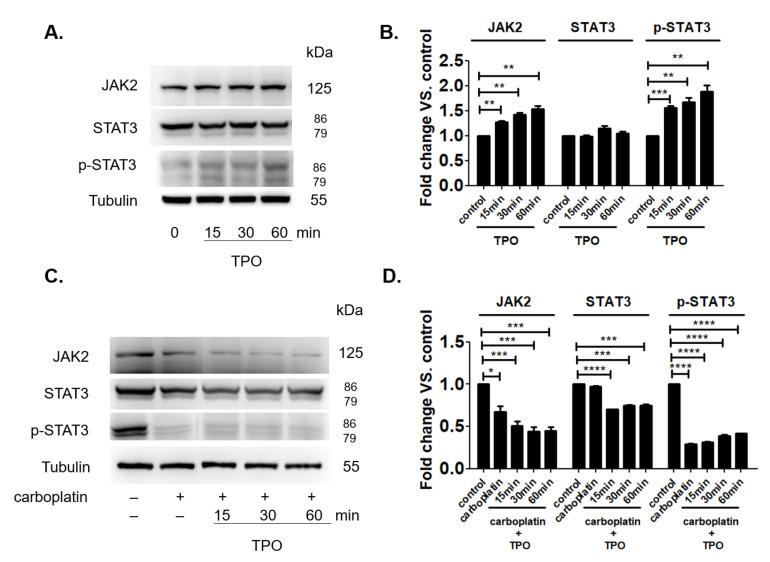
TPO failed to activate STAT3 signal pathway of MEG-01 cells in the presence of carboplatin. (**A**,**B**) MEG-01 cells were treated by TPO for 0–60 min and the expressions of JAK2, STAT3 and p-STAT3 were determined by the western blot method. (**C**,**D**) MEG-01 cells were pretreated with carboplatin for 24 h, then by TPO for 0–60 min and the expressions of JAK2, STAT3 and p-STAT3 were determined by western blot method. (* *p*-value < 0.05, ** *p*-value < 0.01, *** *p*-value < 0.001, **** *p*-value < 0.0001).

TPO can promote the platelet production by binding its receptor on megakaryocytic cells and activate the downstream signal through STAT3 phosphorylation. However, it is not clear why the clinical use of TPO could not exert satisfied efficacy to recover the carboplatin-induced thrombocytopenia [19]. In Figure 1D, we have found that the blood concentration of TPO did not change after the carboplatin treatment for six days in rat when number of platelets was significant lower compared to control. This finding reasonably ruled out the role of TPO in carboplatin-induced thrombocytopenia at the sixth day. Under normal condition, TPO time-dependently increased both JAK2 expression and the activation of STAT3 signal pathway in MEG-01 cells as shown in Figure 6A,B. However, in the presence of carboplatin, TPO failed to increase both JAK2 expression and the activation of STAT3 signal pathway in MEG-01 cells as shown in Figure 6C,D.

## 3. Discussion

The present study, for the first time, reports that carboplatin can induce thrombocytopenia in rat (Table 1 and Figure 1) and reduce the number of megakaryocytes both in vivo and in vitro (Figure 1 and Figure 2, respectively). Carboplatin suppresses the expression of JAK2 in megakaryocytes both in vivo and in vitro (Figure 5) and subsequently inhibits the activation of STAT3 pathway (Figure 5). More importantly, TPO fails to activate JAK2/STAT3 pathway possibly due to the inhibition of c-Mpl and JAK2 in carboplatin-treated megakaryocytes (Figure 6). In addition to the decreased viability of megakaryocytes, this finding deepens the understanding of CIT caused by carboplatin. Although CIT is not an uncommon complication when carboplatin is administered for cancer patients, the exact mechanism of CIT is still an intriguing issue to carboplatin, since it is more prone to cause CIT than other platinum agents [7]. In this study, we found that JAK2 downregulation is highly associated with CIT, which may stress the importance of JAK2 in thrombocytopenia-related diseases. In the recent decades, lots of reports have shown that the crucial roles of JAK2 and TPO in hematopoiesis. However, most studies focus on the role of JAK2 mutation in myeloproliferative diseases (MPD) rather than JAK2 downregulation-associated diseases [17,20]. JAK2 deficient could be barely seen among humans, which may be due to the fact that JAK2 deficiency is lethal in early life [21], and downregulation of JAK2 is mainly mentioned in the therapy for MPD, in which thrombocytopenia becomes a dose-limited factor to the use of JAK2 inhibitors. During the thrombopoiesis process, JAK2 occupies the central role in intracellular signal transduction among megakaryocyte lineage, and only with the intact function of JAK2, TPO can induce megakaryocyte maturation through binding myeloproliferative leukemia (MPL) receptor on megakaryocyte lineage cells [22,23,24]. Insufficient JAK2 may influence the platelet production and function according to the stages of megakaryocyte lineage cells [25,26]. It is found in the early stage of hematopoiesis, such as hematopoietic stem cells (HSC) and colony forming unit-megakaryocytes (CFU-Meg), that JAK2 downregulation is associated with low thrombopoiesis capability. However, thrombocytosis may occur conversely if the JAK2 expression is downregulated in pro-megakaryocytes, mature megakaryocytes, or platelets [25,26]. For this reason, we hypothesized that carboplatin might mainly influence the hematopoiesis in earlier stage since prominent JAK2 downregulation-associated thrombocytopenia is found in our study both in vivo and in vitro. However, further studies are still needed to clarify this issue. 

Moreover, we found that the activation of STAT3 by phosphorylation, as the downstream proteins of JAK2 for thrombopoiesis, were suppressed in megakaryocytes, which may broaden the understanding of pathways involved in CIT, and more importantly, raise the pre-cautions of thrombocytopenia about therapies aiming at JAK/STAT pathway. In previous reports, pathways involved PI3K, caspase and Bcl-2 family proteins for megakaryocytes survival were mentioned as the possible mechanisms of CIT [11,13,27]. The association between decreased megakaryocyte viability and STAT3 activation in CIT found in this study provided the possibility that carboplatin may also influence the renewal ability of megakaryocytes since JAK-STAT pathway is essential to HSC maintenance [28]. The decreased renewal ability, cell viability, and increased apoptosis among megakaryocytes may explain why thrombocytopenia is not uncommon when using carboplatin, and this effect would be initiated even only relatively low dose carboplatin is used as shown in our result on cultured megakaryocytes. The potential risk of thrombocytopenia via suppressing JAK/STAT pathway may also raise the pre-cautions about several ongoing anti-tumor therapies aiming at suppressing this pathway, such as the chimeric antigen receptor therapy containing JAK/STAT domain [29], in which the occurrence thrombocytopenia may become a crucial factor to balance between benefit and risk of treatment. In this case, proper management on CIT would become very important as a complementary therapy. 

Although the occurrence of CIT is not uncommon when treating malignancies, the options of pharmacological treatments of CIT are still limited that only interleukin-11 (IL-11) and thrombopoietin receptor agonists (TRAs) are possible therapies for CIT [1,30,31]. IL-11 is the only one therapy approved by the US Food and Drug Administration (FDA) for CIT, but the use of IL-11 is rarely justified due to the narrow therapeutic index and the considerable side effects, such as edema, fatigue and even conscious disturbance [1,32]. Exogenous TPO administration failed in the clinical practice due to the increased risk of producing neutralizing antibody-related pancytopenia [33]. TRAs, such as romiplostim and eltrombopag, although have not approved by FDA for treating CIT, are becoming the most expected therapies to CIT due to the success in the aplastic anemia (AA) and idiopathic thrombocytopenic purpura (ITP) [34,35]. However, the use of TRAs for treating CIT remains inconclusive to date since there are the only small amounts of clinical trials or observational studies about the efficacy of TRAs on treating CIT [1,19,31]. We suppose that TRAs may not be so effective for treating CIT caused by carboplatin as for AA or ITP since both c-Mpl and JAK/STAT pathway are prominently downregulated after exposure to carboplatin and fails to be re-activated even TPO is administered in our study. Additionally, the markedly high endogenous TPO level under CIT and saturated TPO receptors may also disfavor the use of TRAs for treating CIT [36,37]. Although romiplostim was once reported to relieve thrombocytopenia after multiple carboplatin and radiotherapy in mice [38], the platelet nadir decreased gradually even under romiplostim treatment, which may imply the presence of ongoing megakaryocyte depletion and possible gradually over-saturated TPO receptors. The homeostasis of endogenous TPO depends on the complicated interactions between the expression JAK and c-Mpl on megakaryocyte lineage cells and even platelets [26,39]. In our model, since the influence of platelet could be excluded in the megakaryocyte-only model, we supposed that at least at megakaryocyte level carboplatin may lower the effectiveness of TRAs. The exact targeting cells of romiplostim and the interactions between carboplatin, romiplostim and the entire thrombopoiesis process are worthy of further studies.

In addition to JAK2 downregulation, we also found that carboplatin may cause G1/S-phase cell cycle arrest on megakaryocytes, as previous reports about cell cycle arrest in malignant cells subjected to carboplatin [40]. Indeed, the cell cycle arrest is a necessary step to produce platelets from mature megakaryocytes. The endomitosis process occurs after cessation of megakaryocyte proliferation, and terminal differentiation follows by accumulating cytoplasm and DNA contents [41,42]. Nevertheless, the cell cycle progression is critical in the proliferation of megakaryocyte progenitors [39,43,44]. In this study, we found that the cell cycle arrest of megakaryocytes was highly correlated with decreasing megakaryocyte viability and cell numbers and progressive cell apoptosis, which was more prominent when using high dose carboplatin. This finding implies the presence of pathologic cell cycle arrest during megakaryocyte maturation. The S-phase cell cycle arrest is not helpful in platelet production (platelet counts were low in vivo), but it may make megakaryocytes more prone to apoptosis. Owing to G1/S-phase cell cycle arrest, megakaryocytes may have more difficulties in repairing damage DNA caused by carboplatin and may be more prone to apoptosis due to failure in DNA repair [45]. In addition to DNA damage, the downregulation of JAK/STAT pathway may precipitate the cell cycle arrest [46]. The accumulation of pathologic cell cycle arrested megakaryocytes may be the reason that CIT often occurs with the consecutive lower nadir of platelet count after serial chemotherapy, and long-term cessation of chemotherapy is needed for platelet recovery. This finding also reminds us of the importance of agents to protect megakaryocytes or even accelerate recovery from CIT [31].

We know that the entire course of platelet formation involves different complicated processes. Within the bone marrow, the hematopoietic stem cells first undergo the process of megakaryocyte differentiation and maturation. During maturation, megakaryocytes migrate to sinusoids and form proplatelets. Subsequently, the terminal ends of proplatelets invade through the vessels and release platelets into blood stream. Our study simply focused on investigating the effect of carboplatin on megakaryocytes. Therefore, the current results did not rule out the possibility that other processes of platelet formation may also be suppressed by carboplatin. Another limitation is due to the low amounts of megakaryocytes in bone marrow. Without the purification of megakaryocytes from bone marrow, the quantitative comparison of the JAK2 ex-pression or the cell cycle distribution of megakaryocytes between control and carboplatin-treated rats using bone marrow washed out by western blot analysis or flow cytometer is difficult to reveal the actual molecular change of megakaryocytes in bone marrow. More experiments in the future will be needed to further understand the molecular pathology of carboplatin-induced thrombocytopenia, especially in the bone marrow.

## 4. Materials and Methods

### 4.1. Reagents

Carboplatin was obtained from AbMole BioScience (Houston, TX, USA). Thrombopoietin (TPO) was purchased from Thermo Fisher Scientific Inc. (Waltham, MA, USA). RNase and propidium iodide were obtained from Sigma-Aldrich (St. Louis, MO, USA).

### 4.2. Animal Experiments

Male Sprague-Dawley rats weighted 150–200 g were purchased from BioLasco Taiwan Company (Taiwan) and maintained on a normal rat chow diet for a week before experiments. All animal experiments were approved and performed by the guidelines for the Care and Use of Laboratory by Animal Experiment Committee of the Chang Gung University. Rats were randomly divided into five groups (at least five rats in each group) and sacrificed at 0, 3, 6, 9, and 12 days after intraperitoneal injection of 35 mg/kg carboplatin (0.1 mL normal saline/10 g body weight). Analysis of blood cells was measured by automated hematology analyzer Sysmex XT-1800i, and thigh bones were prepared for histological examination by HE stain and immuno-staining of JAK2 protein. Bone marrows were washed out, fixed and stained by Liu’s stain for the counting of megakaryocytes or CD61+ cells were analyzed by flow cytometry. Bone marrows were also washed out for the analysis of cell apoptosis.

### 4.3. ELISA

The concentration of TPO in rat serum was measured by ELISA kit obtained from Wuhan USCN Business Co., Ltd. Serum collected from rat on the sixth day with or without the carboplatin treatment was used for TPO analysis and 100 µL serum sample from each rat was processed for double-antibody sandwich method according to the method described in manufacturer’s protocol.

### 4.4. Cell Culture

MEG-01 cells were obtained from BCRC (Taiwan) and grown in RPMI 1640 medium supplemented with 10% FBS and antibiotics at 37 °C in a humidified atmosphere with 5% CO_2_/95% air. Cells at a density of 5 × 10^5^ cells/mL were treated with various concentrations of carboplatin for 24 h, and cell viability was determined by trypan blue exclusion assay.

### 4.5. Flow Cytometry

Cells were harvested and centrifuged, resuspended in 1 mL ice-cold 70% ethanol and incubated at −20 °C for 2 h. Cells were collected, washed and incubated in 0.5% Triton X-100 containing 0.05% RNase at 37 °C for 1 h. Cell nuclei were then stained with 50 mg/mL propidium iodide in 1 × PBS and incubated at 4 °C for 20 min. Cell cycle distribution was analyzed using a FACS Calibur system (Becton-Dickinson) and Cell Quest Pro software (Becton Dickinson). The early and late apoptosis of megakaryocytes were determined by using TACS Annexin V-FITC kit purchased from Trevigen. Cells were incubated with the binding buffer containing FITC- conjugated annexin V and propidium iodide for 15 min at room temperature and then the fluorescence was analyzed by flow cytometer. The number of megakaryocytes in bone marrow were identified by incubating the washout in 5% BSA blocking buffer for 30 min and then in buffer containing antibody against CD61 (anti-CD61 #MA1-80862 from ThermoFisher) for 2 h, washing with 1 × PBS, and then in 1 × PBS containing FITC-conjugated secondary antibody for 30 min. The number of megakaryocytes was analyzed by flow cytometer after washing three times in 1 × PBS.

### 4.6. Western Blot Analysis

Cells was solubilized in lysis buffer (20 mM Tris-Cl, pH 7.5, 150 mM NaCl, 1 mM Na2EDTA, 1 mM EGTA, 1% Triton X-100, 2.5 mM sodium pyrophosphate, 1 mM β-glycerophosphate, 1 mM Na_3_VO_4_, 1 mM phenylmethylsulfonyl fluoride, 1 μg/mL aprotinin, and 1 μg/mL leupeptin), followed by centrifugation at 12,000× *g* for 10 min at 4OC. The protein concentrations in the supernatants were determined by Bradford protein assay kit (Bio-Rad, Hercules, CA, USA). Cell lysates containing equal amounts of protein were separated by 10% SDS-PAGE, and transferred onto PVDF membrane (Millipore, Bedford, MA, USA). The membrane was incubated in blocking solution (1% BSA, 1% goat serum in 1×TBST) for 30 min, followed by incubation with properly diluted primary antibody [anti-JAK2 #3230, anti-Stat3 #12640, anti-phospho-Stat3 #9145, Phospho-Chk1 Antibody Sampler Kit #9931, anti-cyclin D1 #2978 from Cell Signaling Technology, Inc. (Danvers, MA, USA); anti-CDK1 #A0220, anti-CDK2 #A2439 from ABclonal,Inc. (Woburn, MA, USA); anti-P21 #MS-387, anti-cyclin A #MS-1061, anti-cyclin B1 #MA1-46103, anti-cyclin E #MA5-14336, anti-tubulin #MS-581-P from Thermo Fisher Scientific Inc. (Waltham, MA, USA); anti-phospho-JAK2 #ab32101 from Abcam; anti-c-Mpl #sc-13187 from Santa Cruz] for 1 h. All of the antibodies were 1:1000 diluted except anti-cyclin E (1:200), anti-c-Mpl (1:200), anti-cyclin A (1:100) and anti-tubulin (1:800). After washing, the membrane was incubated in 1×TBS containing goat anti-mouse or anti-rabbit IgG conjugated with horseradish peroxidase (Sigma-Aldrich, St. Louis, MO, USA) for 1 h. The membrane was washed, and the positive signals were developed with enhanced chemiluminescence HRP substrate reagent (Merck KGaA, Darmstadt, Germany). All of the membrane blotting, washing and signal developing were carried out at room temperature.

### 4.7. RNA Isolation and RT/Real-Time PCR

Total cellular RNA was isolated by Trizol reagent following manufacturer’s protocol. One μg of total RNA was reverse-transcribed into cDNA by incubating with 200 μL of reverse transcriptase in 20 μL of reaction buffer containing 0.25 μg of random primers and first strand cDNA was used for PCR reaction as the template. Primers used in PCR to amplify JAK2 cDNA were s-GCAGT GTTAG ATATGATGAG and as-CTTAT TCGCT TCCTT GTC; to amplify GAPDH cDNA were 5′-CACCTGACCTGCCGTCTA-3′ and 5′-AGGAGTGGGTGTCGCTGT-3′. The SYBR Green method was applied in a Quant Studio 3 qPCR machine (Applied Biosystems by Thermo Fisher Scientific) Each sample was run in triplicate, and GAPDH was used as the internal control for normalization.

### 4.8. Immunohistochemistry

The tissue sections of paraffin-embedded bone marrows were incubated in 60 °C incubator, deparaffinized in xylene and rehydrated through washes with a gradually decreased concentration of ethanol. Slides were washed in 1 × PBS, and antigen retrieval was performed by incubation with antigen retrieval buffer (100 mM Tris, 5% [*w*/*v*] urea, pH 9.5) at 95 °C for 10 min. Sections washed in 1 × PBS were then immersed in 3% H_2_O_2_ for 10 min to reduce endogenous peroxidase activity and then incubated with blocking solution containing 1% BSA and 1% goat serum for 30 min. Sections were incubated with primary antibody [anti-JAK2 #3230 from Cell Signaling Technology, Inc. (Danvers, MA, USA) ] diluted in blocking solution for 2 h. After washing, the sections were incubated in 1 × PBS containing goat anti-rabbit IgG conjugated with PE (Thermo Fisher Scientific, Waltham, MA, USA) for 1 h. After counterstaining the cell nuclei with DAPI, sections were mounted and analyzed by fluorescent microscope.

### 4.9. Statistical Analysis

Results were expressed as mean ± standard error of mean (SEM). Statistical differences were determined by independent and paired Student’s *t*-test in unpaired and paired samples. Whenever a control group was compared with more than one treated group, one-way ANOVA was used instead. Results with a *p*-value ≤ 0.05 were thought statistically significant. Nonparametric tests were used for animal study.

## 5. Conclusions

This study demonstrates that carboplatin can reduce the cell viability of megakaryocytes by inducing S-phase cycle arrest and cell apoptosis. More interestingly, carboplatin can suppress the JAK2 expression and the downstream STAT3 activation which then interferes with the subsequent process of thrombopoiesis. TPO fails to reactivate the JAK2/STAT3 pathway not only due to the fact that JAK2 is suppressed, but also the expression of its receptor, c-Mpl is greatly downregulated by carboplatin. Without the reactivation of JAK2/STAT3 pathway, TPO is unlikely to be effective enough to treat carboplatin-induced thrombocytopenia. Whether other different TRAs can exert therapeutic effect on carboplatin-induced thrombocytopenia through mechanism other than reactivating JAK2/STAT3 pathway needs further investigation in the future.

## Figures and Tables

**Table 1:** Blood cell analysis.

	Control	Carboplatin Day 3	Carboplatin Day 6	Carboplatin Day 9	Carboplatin Day 12	*p*-Value
Weight (g)	214.0 ± 21.3	244.2 ± 54.1	240.3 ± 42.0	223.7 ± 38.2	249.0 ± 57.4	0.28
WBC	5.8 ± 1.6	5.9 ± 1.6	4.8 ± 1.6	5.6 ± 1.9	5.0 ± 1.3	0.56
RBC	6.7 ± 0.5	6.5 ± 0.8	6.4 ± 0.5	6.5 ± 0.6	6.0 ± 1.0	0.25
HGB	12.6 ± 0.5	12.4 ± 0.8	12.2 ± 0.7	12.1 ± 0.9	12.2 ± 1.8	0.53
HCT	41.7 ± 1.1	39.7 ± 2.3	39.9 ± 1.9	39.8 ± 2.8	39.2 ± 5.4	0.16
MCV	62.4 ± 1.9	61.4 ± 5.2	62.2 ± 3.3	61.8 ± 3.1	59.6 ± 3.3	0.51
MCH	18.9 ± 0.6	19.1 ± 1.2	18.9 ± 0.9	18.8 ± 0.8	18.6 ± 1.2	0.87
MCHC	30.3 ± 0.5	31.3 ± 0.9	30.4 ± 0.4	30.5 ± 0.6	31.1 ± 1.0	<0.05
PLT	1117.5 ± 179.7	1142.2 ± 141.4	731.2 ± 141.7	756.7 ± 287.9	982.2 ± 366.2	<0.05
RDW-SD	29.8 ± 1.0	28.7 ± 2.2	29.7 ± 2.2	30.5 ± 2.4	29.9 ± 2.0	0.51
RDW-CV	13.4 ± 0.5	13.4 ± 0.4	13.9 ± 0.8	14.3 ± 0.8	15.1 ± 1.0	<0.05
PDW	7.0 ± 0.5	6.9 ± 0.2	7.0 ± 0.4	7.0 ± 0.8	7.4 ± 1.1	0.66
MPV	6.9 ± 0.4	6.8 ± 0.2	7.0 ± 0.2	7.0 ± 0.5	7.3 ± 0.7	0.37
P-LCR	5.8 ± 1.8	5.3 ± 0.9	6.4 ± 1.5	7.0 ± 2.9	8.4 ± 4.8	0.22
PCT	0.8 ± 0.1	0.8 ± 0.1	0.6 ± 0.2	0.5 ± 0.2	0.7 ± 0.2	<0.05
NEUT	0.7 ± 0.3	0.7 ± 0.2	0.9 ± 0.4	0.9 ± 0.5	0.6 ± 0.3	0.35
LYMPH	4.4 ± 1.4	5.1 ± 1.7	3.6 ± 1.1	4.4 ± 1.7	4.3 ± 1.2	0.61
MONO	0.1 ± 0.1	0.1 ± 0.1	0.0 ± 0.0	0.1 ± 0.1	0.1 ± 0.0	0.18
EO	0.04 ± 0.01	0.03 ± 0.02	0.02 ± 0.01	0.03 ± 0.02	0.04 ± 0.03	0.14
BASO	0 ± 0	0 ± 0	0 ± 0	0.001 ± 0.003	0.002 ± 0.004	0.32

WBC, white blood cell; RBC, red blood cell; HGB, hemoglobin; HCT, hematocrit; MCV, mean corpuscular column; MCH, mean corpuscular hemoglobin; MCHC, mean corpuscular hemoglobin concentration; PLT, platelet; RDW-SD, red blood cell distribution width standard deviation; RDW-CV, red blood cell distribution width coefficient of variation; PDW, platelet distribution width; MPV, mean platelet volume; P-LCR, platelet large cell ratio; PCT, procalcitonin; NEUT, neutrophil; LYMPH, lymphocyte; MONO, monocyte; EO, eosinophil; BASO, basophil.

## Data Availability

All of the data used to support the findings of this study are presented in this article.
